# The role of exercise combined with tocilizumab in visceral and epicardial adipose tissue and gastric emptying rate in abdominally obese participants: protocol for a randomised controlled trial

**DOI:** 10.1186/s13063-018-2637-0

**Published:** 2018-05-02

**Authors:** Regitse Højgaard Christensen, Anne-Sophie Wedell-Neergaard, Louise Lang Lehrskov, Grit Elster Legård, Emma Berndt Dorph, Stine Nymand, Maria Korf Ball, Morten Zacho, Robin Christensen, Helga Ellingsgaard, Jaya Birgitte Rosenmeier, Rikke Krogh-Madsen, Bente Klarlund Pedersen, Kristian Karstoft

**Affiliations:** 1 The Centre of Inflammation and Metabolism (CIM) and The Centre for Physical Activity Research (CFAS), Rigshospitalet, University of Copenhagen, Blegdamsvej 9, DK-2100 Copenhagen, Denmark; 2 Department of Cardiology, Copenhagen University Hospital Bispebjerg, Capital Region of Copenhagen, Copenhagen, Denmark; 3 Musculoskeletal Statistics Unit, The Parker Institute, Bispebjerg and Frederiksberg Hospital, F, Copenhagen, Denmark; 4 Department of Clinical Pharmacology, Bispebjerg Hospital, University of Copenhagen, Copenhagen, Denmark; 5 Department of Rheumatology, Odense University Hospital, Odense, Denmark

**Keywords:** Visceral adipose tissue, Epicardial adipose tissue, Glucose homeostasis, Gastric emptying rate, Interleukin-6, Exercise

## Abstract

**Background:**

Exercise reduces the amount of visceral adipose tissue (VAT) and the risk of cardiometabolic diseases. The underlying mechanisms responsible for these exercise-induced adaptations are unclear, but they may involve lipolytic actions of interleukin-6 (IL-6). Contracting skeletal muscles secrete IL-6, leading to increased circulating IL-6 levels in response to exercise. The aim of this study is to investigate whether IL-6 is involved in mediating the effects of exercise on visceral and epicardial adipose tissue volume and glycaemic control.

**Methods/design:**

Seventy-five physically inactive males and females aged > 18 years with a waist-to-height ratio > 0.5 and/or waist circumference ≥ 88 cm (females) or ≥ 102 cm (males) are being recruited to participate in a 12-week intervention study. Participants are randomly allocated to one of five groups (1:1:1:1:1). Two groups consist of supervised endurance exercise training combined with the IL-6 blocker tocilizumab (ET) or saline used as placebo (EP), two groups consist of no exercise combined with tocilizumab (NT) or placebo (NP), and one group consists of resistance exercise and placebo (RP). Although the study is an exploratory trial, the primary outcome is change in VAT volume from before to after intervention, with secondary outcomes being changes in (1) epicardial adipose tissue, (2) pericardial adipose tissue and (3) gastric emptying. Depots of adipose tissue are quantitated by magnetic resonance imaging Gastric emptying and glucose metabolism are assessed using mixed-meal tolerance tests.

**Discussion:**

Understanding the role of IL-6 in mediating the effects of exercise on visceral and epicardial adipose tissue and glycaemic control may lead to novel therapeutic approaches in the prevention of cardiometabolic diseases.

**Trial registration:**

ClinicalTrials.gov, NCT02901496. Registered on 1 August 2016 and posted retrospectively on 15 September 2016.

**Electronic supplementary material:**

The online version of this article (10.1186/s13063-018-2637-0) contains supplementary material, which is available to authorized users.

## Background

Cardiometabolic diseases are a leading cause of death worldwide and are becoming a dominant health problem globally. Common pathogenic features shared by cardiometabolic diseases are visceral and cardiac adiposity, low-grade inflammation and impaired glycaemic control [1, 2]. It is well known that regular endurance exercise reduces the volume of visceral adipose tissue (VAT) and improves glycaemic control, and also overall lowers the risk of cardiometabolic disease substantially [3–6].

The underlying mechanisms driving the exercise effects on VAT lipid metabolism are poorly understood, but they may involve the cytokine interleukin-6 (IL-6). During intensive exercise, skeletal muscles secrete IL-6 into the circulation [1, 7]. IL-6 has been identified as a lipolytic agent, and a single infusion of recombinant human IL-6 at physiological concentrations increases lipolysis and fat oxidation in humans [8]. This finding was replicated in vitro, with myotubes and adipocytes increasing fat oxidation and lipolysis following IL-6 stimulation [8]. Whether the exercise-induced reductions in VAT are caused by the lipolytic effects of IL-6 or other exercise factors is not known.

Whereas substantial evidence exists regarding the training-induced reductions in VAT, the effect of exercise on cardiac adipose tissue is poorly elucidated. One study has shown that endurance exercise causes a reduction in epicardial adipose tissue thickness [9]. Yet, it is unknown whether the exercise-mediated reduction in visceral fat is equipotent in epi- and pericardial fat (EAT and PAT, respectively). In addition, whether resistance exercise can similarly reduce EAT and PAT volume is unknown. Furthermore, whether the mechanism driving the possible effects of exercise on EAT and PAT involves IL-6 signalling remains to be investigated.

Despite an extensive amount of research on IL-6 in rodents, it remains to be investigated how repetitive systemic spikes of IL-6 (as seen with exercise) affect glycaemic control in humans. On the basis of studies in mice, it is known that IL-6 improves glucose tolerance through a glucagon-like peptide-1 (GLP-1)-mediated increase in insulin secretion [10]. In humans, an acute increase in circulating IL-6 improves glycaemic control by delaying the rate of gastric emptying (in press [11]). In humans, gastric emptying rate is a critical regulator of glycaemic control [12]. Yet, it remains to be investigated whether repetitive systemic spikes of muscle-derived IL-6 influence glycaemic control through changes in gastric emptying rate.

## Methods/design

### Study hypotheses and aims

We hypothesise that endurance exercise plus saline (placebo) is superior in mediating the reduction in visceral, epi- and pericardial adiposity and is superior in delaying gastric emptying compared with endurance exercise plus tocilizumab (IL-6 blocking agent); that is, if the null hypothesis is rejected and there is a difference between the two endurance exercise groups, the alternative hypothesis might be true.

The primary aim of this study is to determine whether blocking IL-6 signalling will ameliorate the endurance exercise-mediated reductions in VAT volume. Secondary aims are to investigate the importance of endurance exercise-induced increases in IL-6 on EAT thickness/area/volume, glucose homeostasis and gastric emptying. Because it is not established if circulating levels of IL-6 are increased in response to resistance training [13, 14], it was not within the scope of this study to include a resistance exercise group receiving tocilizumab. However, as a separate distinctive aim, the resistance exercise plus placebo group was included to investigate the effects of resistance exercise vs. endurance exercise on VAT, EAT and glucose homeostasis.

### Study design and study setting

**Fig. 1:**
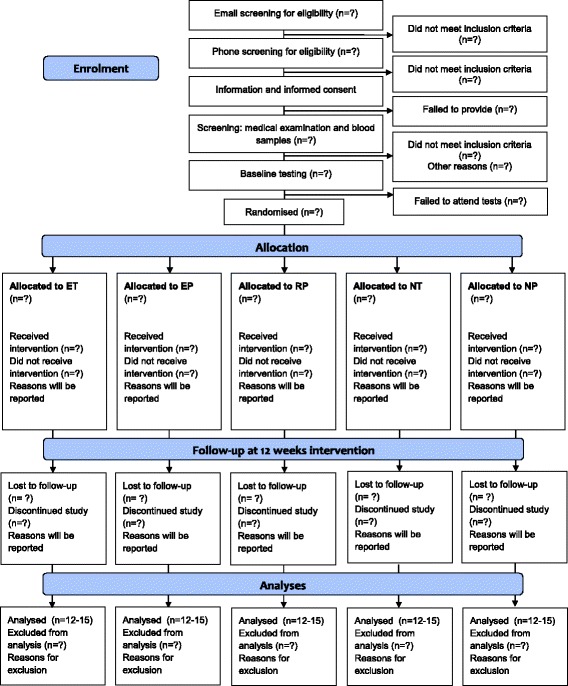
Flow of participants through the study. Any reasons for discontinuation or exclusion from analysis are reported. *ET* (Endurance exercise + tocilizumab), *EP* (Endurance exercise + saline (placebo)), *RP* (Resistance exercise + saline), *NT* (No-exercise control + tocilizumab), *NP* (No-exercise control + saline)

The study is designed as an exploratory, double-blind, randomised, placebo-controlled trial consisting of 12 weeks of endurance exercise (E) or no exercise (N) combined with monthly infusions of tocilizumab (T) or placebo (P), resulting in five groups (ET, EP, NT, NP and RP), where RP is a placebo group using resistance exercise rather than endurance exercise. A total of 75 physically inactive participants are randomised in a ratio of 1:1:1:1:1 to exercise groups or control groups in combination with tocilizumab or saline, as depicted in Fig. 1. The enrolment period began on 1 August 2016 and was estimated to be completed by 26 April 2018. All baseline and follow-up tests are carried out at the Centre for Physical Activity Research (CFAS), Rigshospitalet, Copenhagen, Denmark, and the MRI department of Frederiksberg Hospital, Frederiksberg, Denmark.

### Participants

#### Eligibility

Participants are recruited through advertisements in local newspapers and via social media. Eligibility criteria are listed in Table 1. Initial inclusion and exclusion criteria are identified through email and phone interview. Participants are eligible if they are white, aged 18–80 years, abdominally obese and physically inactive (Table 1). Participants of same race are chosen in order to limit the effect of racial background on lipid and glucose metabolism [15]. The broad age span is chosen because IL-6 response to exercise is not known to be age-dependent [16, 17]. Abdominal obesity, which is indicative of visceral adiposity, is defined as a waist circumference > 88 cm for women or 102 cm for men [18, 19]. Physical inactivity is defined as no engagement in any intentional leisure exercise activities and failure to meet the current American College of Sports Medicine (ACSM) exercise guidelines of 2.5 h/wk [20, 21] by unintentional physical activity associated with daily activities. The unintentional daily activity level is evaluated individually at the telephone screen by asking a number of questions, including (1) type of occupation, which should be sedentary (defined by primarily computer-based activities [20, 22]); (2) way of commuting, which should preferably be passive or alternatively biking at a slow pace < 30 min/d on a maximum of 5 d/wk; and (3) the total daily activity level assessed by steps per day should be < 10,000, which is a reliable threshold for meeting current activity guidelines [23]. If eligible after the phone interview, the participant provides informed oral and written consent and subsequently undergoes a medical screening that includes blood sampling in order to identify latent exclusion criteria.

**Table 1:** Eligibility of study participants

Inclusion criteria	Exclusion criteria
1. Age 18–80 yr 2. White race 3. Physically inactive (defined as not meeting the current guidelines of 2.5 h of physical activity per wk) 4. Waist-to-height ratio ≥ 1:2 and/or waist circumference ≥ 88/102 cm (for women/men) 5. Permitted medicine should be taken at a stable dose during at least 4 wk prior to randomization and preferably remaining stable during the study.	1. Diabetes (HbA1c ≥ 48 mmol/mol or fasting glucose ≥ 7.0 mmol/L) 2. Pregnancy 3. Ischaemic heart disease 4. Atrial fibrillation 5. Treatment with: a. NSAIDs on a daily basis b. Biologic rheumatic drugs c. Systemic prednisolone d. Other immunotherapy 6. Health conditions that prevent individuals from participating in the exercise training intervention 7. Subjects who cannot undergo MRI scans (e.g., owing to kidney disease, metallic implants or claustrophobia)

*Abbreviations*: *HbA1c* Glycated haemoglobin, *NSAIDs* Non-steroidal anti-inflammatory drugs, *MRI* Magnetic resonance imaging

### Interventions

The intervention is a combination of two components: a medical intervention (tocilizumab [T] or placebo [P]) and a lifestyle intervention (supervised exercise [E] or no [N] exercise) as detailed below. The resulting intervention and control groups are depicted in Fig. 1.

#### Medical intervention: IL-6 antagonism (tocilizumab)

Tocilizumab (RoActemra®; Roche, Basel, Switzerland) is a humanised monoclonal antibody against the IL-6 receptor currently used for treatment of patients with moderate to severe rheumatoid arthritis [24]. Participants will receive an intravenous infusion of 8 mg/kg (maximal 800 mg) administered every 4 weeks during the 12-week intervention (a total of three times) [25]. We chose to give the maximal recommended dose of tocilizumab [24] because we aimed for maximal suppression of the effects of exercise-induced IL-6 secretion. Tocilizumab is generally well tolerated. Serious adverse reactions are rare but include infections and hypersensitivity reactions. Tocilizumab is purchased via the pharmacy at Rigshospitalet. It is prepared according to the recommendation (8 mg/kg) and diluted in NaCl 0.9% to a total volume of 100 ml. Qualified staff at CFAS are responsible for this procedure. The placebo consists of 100 ml of NaCl 0.9%. Both placebo and tocilizumab are administered via intravenous infusion at a flow rate of 2 ml/min, and the infusion is concealed to maintain double blinding.

#### Exercise intervention and lifestyle monitoring

The exercise programme includes three weekly training sessions of 45 min/session over a 12-week period. Each session will be moderate to vigorous in intensity. The exercise programme follows current ACSM guidelines for vigorous exercise [21] and was chosen because exercise of similar intensity and duration is known to result in IL-6 release [1, 17] and lead to changes in VAT (the primary outcome) [13]. Training takes place at the Centre for Physical Activity Research CFAS using TechnoGym equipment (Pedan A/S, Copenhagen, Denmark) and software programs which allow monitoring and registration of all exercise sessions. All exercise sessions are supervised. Maximal oxygen consumption rate (VO_2_ max) and one-repetition maximum (RM) of two resistance exercises (knee extension and chest press) are determined at baseline and after the intervention for each participant. To control for factors that may influence the effect of the interventions, participants are instructed not to change their lifestyle during the intervention, with the exception of the intervention. A self-reported 3-day dietary intake record is obtained monthly during the intervention period. Furthermore, free-living physical activity is measured monthly using axial accelerometer-based physical activity monitors (AX3; Axivity, Newcastle upon Tyne, UK) for a 4-day period. The monthly registrations are undertaken in relation to pre- and mid-testing as well as at post-testing on identical visits and on identical consecutive weekdays throughout the intervention to ensure comparable measurements.

#### Endurance exercise

**Fig. 2:**
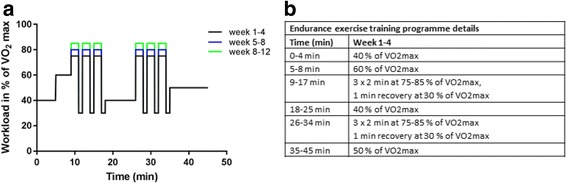
Protocol of the endurance exercise. **a** Graphic demonstration of the different workload intervals of the endurance exercise programme. The workload is defined by the corresponding maximal oxygen consumption rate (VO_2_ max) percentage. **b** Details of the exercise programme

High-intensity interval endurance exercise is performed on an ergometer bike. The intensity of the intervals progressively increases during the 12 weeks of training. The 45-minute exercise sessions consist of 8 minutes of warm-up at 40% and 60% of VO_2_ max followed by 37 minutes of interval exercise at 75–85% of VO_2_ max, as shown in Fig. 2.

#### Resistance exercise

**Fig. 3:**
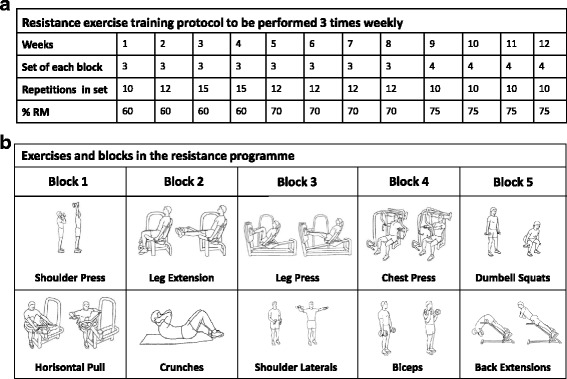
Overview of the resistance exercise protocol. **a** Progression in frequency of sets and repetitions during the 12-week exercise intervention performed 3 × weekly. **b** The ten individual exercises that are paired in five blocks and performed in each resistance exercise session with a duration of 45 min/session

The resistance exercise is matched to the endurance training with a duration of 45 minutes per session. The exercise is interval-type, medium-load, high-repetition, time-based resistance exercise. Participants perform ten exercises paired in five blocks. Three to five sets with ten to fifteen repetitions lasting 1 minute are performed. The aim is to reach complete exhaustion after each exercise. The specific resistance exercise load comprises 60–80% of 1 RM with progression during the 12 weeks. Ten different exercises are performed: shoulder press, leg extension, pull exercise, crunches, leg press, shoulder laterals, chest press, biceps, dumbbell squats and back extensions. Details of the resistance exercise protocol are depicted in Fig. 3.

### Outcomes

Outcome variables are listed in Table 2.

**Table 2:** Outcome domains and measures assessed at baseline and follow-up (12 weeks after baseline)

Outcome	Domain	Measurement
Primary	Visceral fat tissue volume	MRI
Secondary	Epicardial adipose tissue thickness/area/volume	Cardiac MRI
	Pericardial adipose tissue thickness/ area/volume Gastric emptying rate	Cardiac MRI MMTT and PCM
Other	Total body fat mass	DXA
	Total body muscle mass	DXA
	Resting blood pressure	BP monitor
	Heart rate recovery	Heart rate strap
	Maximal aerobic capacity (cardiovascular fitness)	VO_2_ max test
	Muscle strength	One-RM test
	Diabetes, pre-diabetes and glucose tolerance	OGTT
	Glycaemic control during mixed-meal tolerance test	MMTT
	Free-living glycaemic control	CGM
	Acute exercise bout^a^	Blood tests
	Low-grade inflammation^b^	Blood tests
	Cardiometabolic biomarkers^c^ and adipokines	Blood tests

*Abbreviations*: *CGM* Continuous glucose monitoring, *DXA* Dual-energy X-ray absorptiometry, *MMTT* Mixed-meal tolerance test, *MRI* Magnetic resonance imaging, *OGTT* Oral glucose tolerance test, *PCM* Paracetamol, *RM* Repetition maximum

^a^Blood will be analysed for exercise factors, including interleukin-6, immune cell counts and function, and biomarkers of low-grade inflammation

^b^Blood will be analysed for biomarkers of low-grade inflammation, including high-sensitivity C-reactive protein, tumour necrosis factor-α, interferon-γ and interleukins

^c^Blood will be analysed for cardiometabolic biomarkers, including total cholesterol, high-density lipoprotein, low-density lipoprotein, glycated haemoglobin, fasting insulin, fasting glucose and pro-brain natriuretic peptide

#### Primary outcome

The primary outcome is the change in volume of VAT (measured using MRI) from baseline to after the 12-week intervention period, with comparison of the groups (Fig. 1).

#### Secondary outcomes

The secondary outcomes are changes in (1) EAT, (2) PAT and (3) gastric emptying. Also, we will explore the effects related to the interventions in regard to changes and alterations in rate of glucose homeostasis and low-grade inflammation.

### Measurement of outcome variables

**Fig. 4:**
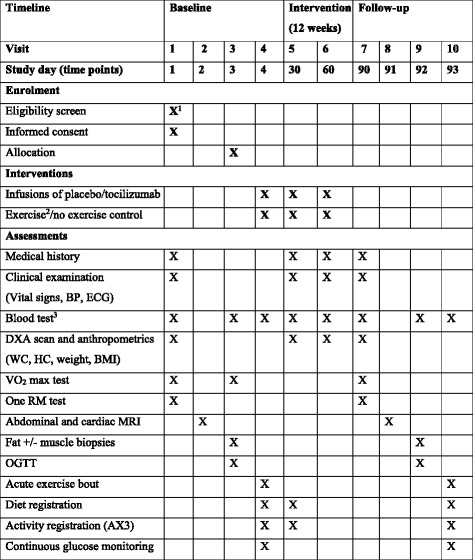
Time schedule of enrolment, interventions and assessments. *Abbreviations: BMI* Body mass index, *BP* Blood pressure, *DXA* Dual-energy X-ray absorptiometry, *ECG* Electrocardiogram, *MRI* Magnetic resonance imaging, *HC* Hip circumference, *VO*_*2*_ *max* Maximal oxygen consumption rate, *WC* Waist circumference, *MMTT* Mixed-meal tolerance test, *OGTT* Oral glucose tolerance test, *RM* Repetition maximum. ^1^Eligibility screen was conducted in a telephone interview before study day 1. ^2^The exercise intervention is a 12-week supervised exercise programme with three sessions per week consisting of either endurance or resistance exercise training. ^3^Blood samples will be drawn after an overnight fast (> 10 hours) and analysed for haematologic, renal, endocrine, cardiac and hepatologic markers. Table is adapted from the SPIRIT recommendations [38]

The time points at which the outcomes are assessed at baseline, during the intervention and at follow-up are depicted in Fig. 4. The study is initiated with a clinical examination followed by baseline testing (Fig. 4, visits 1–4). Monthly tocilizumab/saline (placebo) infusions (Fig. 4, visits 4–6) are administered during the intervention period. After the intervention period (12 weeks), the participants undergo follow-up testing (Fig. 4, visits 7–10) identical to the baseline tests.

#### Blood samples

Following an overnight fast (10 hours), blood samples, as specified in Fig. 4, are collected and processed by a trained laboratory technician and analysed according to standard procedures. Plasma is stored at − 80 °C prior to analysis.

#### Abdominal and cardiac magnetic resonance imaging

MRI is performed at Frederiksberg Hospital using a 1.5-Tesla whole-body MRI scanner (Ingenia; Philips, Eindhoven, the Netherlands). Abdominal MRI scans are obtained as transverse images (slice thickness of 5 mm) from the top of the thoracic diaphragm to the pelvic floor. VAT will be manually marked on each image, capturing the area from the diaphragm to the first sacral vertebra, and the volume will be calculated as the sum of all voxels using MANGO (Multi-Image Analysis GUI) version 2.5 [26, 27].

Cardiac MRI is supervised by a senior cardiologist, and sagittal, axial and horizontal planes are obtained from five retrospective cardiac cycles. Cardiac function including myocardial mass is quantified using cine imaging with a slice thickness of 8 mm (inter-slice gap, 2 mm for the short-axis stack) and a temporal resolution of 30–50 milliseconds, depending on heart rate (25 acquired phases). The number of short-axis cine images acquired is participant-specific, depending on ventricular size, using retrospective gating when possible in all participants. Semi-automatic calculations using cardiovascular imaging software (cvi^42^ version 5.2; Circle Cardiovascular Imaging, Calgary, AB, Canada) will be used for post-processing using endo- and epicardial contours to calculate left ventricular ejection fraction and cardiac muscle mass. EAT and PAT thicknesses, areas and volumes will be quantified from images in the end-diastolic four-chamber view and short-axis view. Contours around the fat layers of the ventricles and atria will be drawn manually. All MRI scans will be analysed in a blinded manner by trained medical doctors. A subset of the cardiac MRIs will be evaluated by a cardiologist to assess accuracy and inter-reader variability.

#### Maximal aerobic capacity

The participants undergo a graded exercise test on an ergometer bicycle for determination of VO_2_ max. The test starts with a 5-minute warm-up at 70 W. Warm-up is immediately followed by a 15-W increase every minute until exhaustion. Ventilation rate and expired O_2_ and CO_2_ levels are measured via an indirect calorimetric system, and heart rate is assessed simultaneously for the duration of the test. Two separate VO_2_ max tests are performed during the baseline tests (Fig. 4) to allow participants to familiarise themselves with the bicycle and the indirect calorimetric ventilation system. The test with the best performance is used to assess the changes in aerobic capacity from baseline to follow-up.

#### Body composition

Dual-energy x-ray absorptiometry is used to assess total body fat mass and lean body mass one time before, two times during and one time after the intervention.

#### Mixed-meal tolerance test

The mixed meal consists of a 350-ml liquid drink containing 385 kcal. The energy distribution is 15% fat, 20% protein and 65% carbohydrate (of which 9% is glucose). To allow assessment of the rate of gastric emptying, 1.5-g paracetamol is dissolved and added to the meal as a pharmacological tracer [28]. Participants are encouraged to ingest the meal in less than 2 minutes, and serial blood samples will be drawn − 10, 0, 15, 30, 60, 90, 120, 150 and 180 minutes after intake of the liquid meal. In addition to paracetamol, blood samples will be analysed for glucagon, GLP-1, GIP, PYY, insulin, pro-insulin, C-peptide and glucose.

#### Oral glucose tolerance test

A standard 2-hour 75-g oral glucose tolerance test is performed following an overnight fast (10 hours). Blood samples are drawn at baseline (0), 15, 30, 60 and 90 minutes and after 120 minutes, and then they are analysed for glucose, insulin, and C-peptide.

#### Continuous glucose monitoring

Three days of continuous glucose monitoring (CGM) is performed using enzyme-coated electrodes (iPro MMT-7745WW; Medtronic, Northridge, CA, USA) placed subcutaneously in the abdominal wall. For calibration of the CGM system, finger-prick blood glucose measurements are performed by the participant four times daily.

#### Acute bout of exercise

For the acute bout of exercise, participants perform a 45-minute exercise bout similar to an exercise session. The workload is determined according to the latest individual watt-VO_2_ relationship assessed during the pre- and post-VO_2_ max test. Blood samples are drawn prior to, during and after the exercise bout (baseline and at 22, 45, 105 and 165 minutes) and analysed for IL-6, immune cell count and function, and other plasma biomarkers.

### Statistical power and sample size considerations

For a two-sample pooled *t* test of a normal mean difference with a two-sided significance level of 0.05, assuming a common SD of 265-g VAT, a sample size of 14 in each group has a power of 82.2% to detect a difference between the means − 300 and 0 g VAT in the endurance exercise group + saline group (EP) and the endurance exercise + tocilizumab group (ET), respectively. The expected change in VAT is based on unpublished data from the CFAS. Fourteen participants will also be included in each of the control groups (NT and NP) and the resistance exercise + saline group (RP). Anticipating only a minor risk of attrition (over 12 weeks), a final sample size of 75 participants will be included in the study (anticipating 15 in each group).

### Analysis populations

This study (2TRAIN) primarily follows a per-protocol design where participants are included in the analysis only if they have completed the trial and adhered to tocilizumab/placebo combined with exercise/no-exercise intervention according to the pre-specified protocol. This design was chosen because we are interested in measuring the physiological effect which is not influenced by lack of adherence, unlike the intention-to-treat (ITT) principle (with use of “conservative imputation”) [29]. Using the per-protocol design enables us to assess the true experimental effect of the medical and exercise intervention on the primary endpoint [29]. Because non-adherers generally differ from adherers with regard to motivational and prognostic factors, this introduces a bias in the per-protocol design. Yet, this bias is limited because the aim of this study is exploratory, with the aim of assessing the mechanism driving the exercise effects, which requires adherence rather than evaluation of treatment efficacy where differential prognostic factors would confound the effect measures.

Patterns of non-adherence and other missing data will be investigated but will be assumed to be missing at random. In case of high non-adherence, high drop-out rate or skewed drop-out between intervention groups, a third-party biostatistician will perform analyses that adjust for such instances. Satisfactory adherence is defined as complete adherence to tocilizumab/placebo intervention and a minimum of 80% adherence to the exercise intervention/no-exercise control.

In addition, the ITT principle will be applied when necessary for the purpose of sensitivity analysis to assess the robustness of the primary analyses where imputation techniques (e.g., baseline observations carried forward) will be used to replace missing data [30]. Any discrepancies in data analysis between the methods will be resolved by a third-party biostatistician.

### Statistical analyses and reporting

Like all clinical trials, exploratory studies should have clear and precise objectives. However, in contrast to confirmatory trials, the objectives of the exploratory 2TRAIN trial may not lead to simple tests of predefined hypotheses; that is, we will apply a more flexible approach to the statistical interpretation of the findings. As a consequence, a trial such as 2TRAIN cannot be the basis of a formal proof of efficacy, although it may contribute to the total body of relevant physiological evidence.

The results will be reported in accordance with the Consolidated Standards of Reporting Trials (CONSORT) [31, 32]. Descriptive variables will be summarised by means of frequency distributions, means and SDs, or medians and IQRs, by group as well as by the total population. Categorical data will be summarised by numbers and percentages.

The primary null hypothesis is based on the comparison between participants randomised to endurance exercise with (ET) or without (EP) tocilizumab; H_0_: μ_ET_ = μ_EP_. The overall study design is based on five groups (EP, ET, NT, NP and RP), and rather than looking only at the two according to the primary objective, we apply linear modelling based on generalised linear models (GLMs) in the analysis of the primary outcome. Analysis of covariance (ANCOVA) models will be performed, which produces several diagnostic measures, provides contrasts and estimates for customised hypothesis tests, and it provides tests for means adjusted for covariates (e.g., least squares [LS] means adjusted for baseline variables). The GLM procedure handles models relating a continuous dependent variable to one or several independent variables. The independent variables can be either classification variables, which divide the observations into discrete groups (e.g., EP, ET, NT, NP and RP), or continuous variables (e.g., the level at baseline). We will analyse continuous outcomes using ANCOVA models, with a fixed factor for group (five levels) and adjust for the value at baseline. For each continuous outcome variable (after performing the overall ANCOVA model [five-group comparison]), we will obtain the *P* value and group contrast for the difference between the LS means: ET vs. EP (in analogy to a two-sample *t* test [33]), independent of what the overall ANCOVA model indicates. Also, results following the ANCOVA model will be expressed as estimates of the group difference in the various pairwise comparisons (including ET vs. EP) in the changes from baseline, with 95% CIs to represent the precision of the estimates. To assess the adequacy of the linear models describing the observed data, as well as to check the assumptions for the systematic and random parts of the models, we will investigate the model features via the predicted values and the residuals; that is, the residuals have to be normally distributed (around 0) and be independent of the predicted values.

All reported *P* values will be two-sided and will not be adjusted for multiple comparisons (despite having ten potential tests for each outcome variable [5 × {5 − 1}/2]). Per default, we set the statistical significance at the conventional level of 0.05 (*P* < 0.05). All analyses will be performed using commercially available statistical software.

#### Blinding

The primary investigators are blinded to intervention. Outcome assessors are blinded to allocation at baseline and follow-up. Owing to the nature of the study, participants cannot be blinded to the exercise modality, but they are blinded to tocilizumab intervention. Interim analyses are not performed.

#### Randomisation: sequence generation and allocation concealment

Participants are randomly assigned to groups in a 1:1:1:1:1 ratio using computer-generated block randomisation. Seven blocks of ten participants and a final block of five participants were planned. In order to achieve a balanced sample size of complete cases in each group, drop-outs occurring in the first six blocks were replaced in the subsequent block. If drop-outs are encountered in the seventh and eighth blocks, these are not replaced. The randomised sequence generation was generated by a researcher who is not involved in the testing and was delivered concealed to the technical personnel preparing the infusions. Infusions are delivered to the study investigators in a concealed manner to maintain blinding. The allocation information regarding exercise modality is given to the participants by a study assistant not involved in the randomisation or evaluation procedures. Exercise sessions are supervised by educated study assistants to maintain blinding of primary investigators with regard to exercise modality.

### Safety (based on collected adverse events)

Before every infusion of tocilizumab/placebo, participants undergo a clinical examination and an interview performed by a medical doctor to assess health status, followed by collection of a blood sample to detect any adverse reactions (including drug-related side effects). The following parameters are measured: alanine aminotransferase, aspartate aminotransferase, creatinine, haemoglobin, thrombocytes, glucose, total cholesterol, low-density lipoprotein cholesterol, high-density lipoprotein cholesterol, triglyceride, high-sensitivity C-reactive protein, leucocytes and leucocyte types.

Intravenous infusion of gadolinium during cardiac MRI scan can rarely cause adverse allergic reactions, but the medical doctors present are educated to identify and handle such situations. Rarely, infections or temporary paraesthesias occur in the skin lesion that follows blood sampling or muscle/fat biopsies. Participants are informed about side effects and signs of infection. The participants are urged to contact the medical doctors involved in the trial at all times in case of an adverse reaction or other symptoms experienced during the study period. Any serious adverse event (life-threatening, fatal, expected, unexpected, or drug-related) will be reported to the scientific ethics committee at the Capital Region of Denmark and, if requested, to the Danish Medicines Agency.

## Discussion

In this study, we are investigating the role of IL-6 in mediating exercise-induced adaptations in an abdominally overweight population. We anticipate the results to provide insight into the role of IL-6 in exercise-mediated changes in VAT, EAT and PAT and glucose homeostasis. Furthermore, we hope to gain valuable insight related to the exercise modalities that can be translated into efficient exercise programmes for subjects with increased risk for metabolic diseases. Understanding how exercise and IL-6 regulate VAT, EAT and PAT volumes and glucose homeostasis may inform novel therapeutic approaches in the prevention and treatment of cardiometabolic diseases.

The study is a randomised controlled trial, and both participants and primary investigators are blinded with respect to the medical intervention of tocilizumab and the outcome assessments. It is not possible to blind participants with respect to the exercise intervention, and this may introduce a bias. To limit this bias, participants are urged not to change their lifestyle during the intervention, except from what they are randomised to, because this would potentially influence the outcomes of the intervention. The eating habits and activity levels of the participants are regularly assessed by use of dietary records and axial accelerometer-based physical activity monitors. It is important to acknowledge that the resistance and endurance exercise protocols are matched only in duration. This is a limitation which should be kept in mind when interpreting the effects of the different exercise modalities. To compare the intensity of the different exercise modalities, we ask the participants to wear a heart rate monitor during both resistance and endurance exercise sessions. Exercise intensity is determined by percentage of VO_2_ max, and not by the peak power output, in line with previous studies by our group [34, 35]. Helgerud et al*.* [36] previously showed VO_2_ max to increase in response to high-intensity interval exercise training. Therefore, we believe that adjustment of exercise intensity according to percentage of VO_2_ max accurately ensures high exercise intensities throughout the intervention in this study. In addition, the supervised nature of the exercise protocol will ensure participant adherence and intensity and thereby improve the homogeneity of each intervention. The measurement of the primary outcome by MRI follows the gold standard and will enable accurate estimations of changes in the VAT, EAT and PAT depots.

The study includes five intervention groups (ET, EP, NT, NP and RP), and the endurance exercise groups with and without tocilizumab (ET vs. EP) are of primary interest. In order to control for isolated effects of tocilizumab, two no-exercise control groups (NT and NP) were included as well. The inclusion of a resistance exercise group without tocilizumab (RP) was intended only for future ad hoc objectives, including comparisons of exercise modalities (endurance vs. resistance exercise without tocilizumab) on changes in VAT, EAT, PAT and gastric emptying.

This study is conducted in accordance with the guidelines of the regional ethics committee and the Declaration of Helsinki II [37]. All participants receive written and oral information about the study prior to inclusion and their provision of oral and written consent. The participants can discontinue participation in the study at all times with no obligation to provide a reason. Data and associated biological material are stored in an individual database and research biobank at Rigshospitalet. The biological material will be destroyed after a maximum of 10 years. Confidentiality of the participants is maintained by assigning participants a study number, keeping identifiers separate from the data, and storing data in a locked file and secure computer database. Scientific reports generated from the study will not contain information that would identify the participants.

The study protocol adheres to the Standard Protocol Items: Recommendations for Interventional Trials (SPIRIT) guidelines [38] (Additional file 1), and results of this study will be reported according to the CONSORT guidelines [31]. Negative, positive or inconclusive results will be disseminated in international peer-reviewed scientific journals at national and international conferences.

## Trial status

The study was prospectively registered at ClinicalTrials.gov (NCT02901496) on 1 August 2016 and posted retrospectively on 15 September 2016. The recruitment period began on 1 August 2016 and was estimated to be completed by 26 April 2018.

## Additional file

Additional file 1: SPIRIT 2013 checklist: recommended items to address in a clinical trial protocol and related documents. (DOC 131 kb)

## Abbreviations

ACSM: American College of Sports Medicine
ANCOVA: Analysis of covariance
BP: Blood pressure
CFAS: Centre for Physical Activity Research
CGM: Continuous glucose monitoring
CONSORT: Consolidated Standards of Reporting Trials
DXA: Dual-energy x-ray absorptiometry
EAT: Epicardial adipose tissue
ECG: Electrocardiogram
EP: Endurance exercise training and placebo
ET: Endurance exercise training combined with tocilizumab
GLM: Generalised linear model
GLP-1: Glucagon-like peptide-1
HbA1c: Glycated haemoglobin
HC: Hip circumference
IL-6: Interleukin-6
ITT: Intention to treat
LS: Least-squares means
MMTT: Mixed-meal tolerance test
MRI: Magnetic resonance imaging
NP: No exercise combined with placebo
NSAID: Non-steroidal anti-inflammatory drug
NT: No exercise combined with tocilizumab
OGTT: Oral glucose tolerance test
PAT: Pericardial adipose tissue
PCM: Paracetamol
RM: Repetition maximum
RP: Resistance exercise and placebo
SPIRIT: Standard Protocol Items: Recommendations for Interventional Trials
TNF-α: Tumour necrosis factor-α
VAT: Visceral adipose tissue
VO_2_ max: Maximal oxygen consumption rate
WC: Waist circumference
